# Changes in Aroma Profile of Shiitake Mushroom (*Lentinus edodes*) during Different Stages of Hot Air Drying

**DOI:** 10.3390/foods9040444

**Published:** 2020-04-07

**Authors:** Lei Qin, Jing-Xuan Gao, Jia Xue, Dong Chen, Song-Yi Lin, Xiu-Ping Dong, Bei-Wei Zhu

**Affiliations:** School of Food Science and Technology, National Engineering Research Center of Seafood, Collaborative Innovation Center of Seafood Deep Processing, Dalian Polytechnic University, Dalian 116034, China

**Keywords:** volatile compounds, electronic nose, PT-GC-MS, fingerprinting, Shiitake mushroom

## Abstract

To clarify the changes in the aroma characteristics of shiitake mushrooms (*Lentinus edodes*) during hot-air drying, volatile compounds of *L. edodes* were analyzed using sensory evaluation, electronic nose, and purge and trap combined with gas chromatography-mass spectrometry (PT-GC-MS) at different timepoints of the drying process. Results showed that the sensory and volatile profile changed significantly during the drying process at 60 °C for up to 12 h and the drying process could be divided into three stages: early stage (<2 h), middle stage (2–3.5 h) and late stage (>3.5 h). Volatile compounds in fresh *L. edodes* consisted mainly of ketones and alcohols. The early stage of drying decreased the concentration of ketone and alcohol compounds and promoted the generation of cyclic organosulfur compounds through a series of enzymatic and non-enzymatic reactions, which mainly contribute to the characteristic odor of shiitake mushroom. Partial least squares-discriminant analysis (PLS-DA) showed that the volatile compounds released after different drying times could be divided into four groups, which have been confirmed by sensory evaluation results. The results suggested that the unique flavor of dried mushrooms is mainly due to the activation of enzymes during the drying process, which act on lentinic acid to produce sulfur-containing heterocyclic compounds. We believe that our study makes a potential contribution to the mushroom cultivation and processing industry to achieve an improvement in sensory quality.

## 1. Introduction

Shiitake mushrooms (*Lentinus edodes*) are one of the most popular edible mushrooms around the world, particularly appreciated for their nutritional and flavor properties [1]. Shiitake is the world’s second largest mushroom and regarded as “the Queen of mushrooms”. The increasing popularity and consumption of *L. edodes* underlines the need to evaluate the quality of the food products available commercially [2]. However, during post-harvest storage, *L. edodes* is sensitive to saturated humidity: high relative humidity with some temperature fluctuation can cause moisture to congeal on the mushroom’s surface, favoring microbial growth and deterioration, and consequently reducing shelf life [1]. Drying technologies play a major role in food manufacturing and food processing activities worldwide and are a cheap method to extend the shelf life of fruits and vegetables by reducing moisture content [3]. Drying can prevent the growth of microorganisms, delay the activity of enzymes, and delay degradative reactions which utilize water as a medium [4]. Shiitake mushrooms are often dried for preservation, and this process causes distinctive hedonic properties of the product. For example, when shiitake mushrooms are dried, the unique shiitake aroma is developed, and 5′-guanosine monophosphate and vitamin D are found in higher concentrations than in fresh mushroom, thus enhancing their taste and nutritional value [5]. People enjoy the unique flavor of dried mushrooms [4]. Some studies have shown that *L. edodes* drying can promote the production of volatile substances not found in the fresh product [6]. However, public information related to the dynamic changes in sensory attributes and volatile compounds during hot air drying is limited.

Sensory evaluation is a conventional method used to evaluate food flavor [7]. In this study, sensory evaluation was used to monitor *L. edodes* flavor changes at different drying times. However, sensory evaluation is not always an objective judgment and may be swayed by physical or environmental factors [8]. Other technologies, as the electronic nose and Gas Chromatography-Mass Spectrometry (GC-MS), should therefore also be used to evaluate *L. edodes* flavor changes at different drying times.

The electronic nose comprises an array of electronic chemical sensors with partial specificity and an appropriate pattern-recognition system. The detection of volatiles is performed using an array of gas sensors. Mixtures of volatiles can be identified, after a training stage, by using the pattern generated from the sensors. The successful use of the electronic nose to differentiate the geographical origin or variety of agricultural products has been well-documented for products such as coffee, tea, wine, fish, and meat [9,10,11]. However, there were no reports about the monitoring of different drying stage of shiitake mushrooms.

The purge and trap (PT) technique is an advantageous extraction method for many aroma compounds, especially those with low boiling points [12]. Additionally, by using this technique, it is possible to extract volatile compounds without artifact formation, then using gas chromatography (GC) together with mass spectrometry (MS) for reliable quantification and identification of volatile compounds [13]. The PT technique is more suitable than SPME for the extraction of low molecular weight compounds and their quantitation. Likewise, PT is advantageous in analyzing large numbers of samples, firstly because of the relatively low cost and amount of solvents involved and, secondly because of the capability of extracting several samples simultaneously [14].

While the electronic nose offers synthesized describers of aroma characteristics, GC-MS offers profiles of the chemical aroma ingredients. The previous literature on the degree of correlation between the electronic nose and GC-MS data is limited. The aims of this paper were to evaluate and discuss the overall aroma of and volatile profile changes in *L. edodes* during hot air drying by sensory evaluation, electronic nose, and PT-GC-MS analyses. Furthermore, the established aroma profile fingerprinting method could be potentially used for an easy determination of product quality.

## 2. Materials and Methods

### 2.1. Chemicals and Materials

Fresh *L. edodes* were picked from Dalian, Liaoning, China. The pileus of fresh *L. edodes* was removed manually. The fresh stripes of *L. edodes* were cut into uniform pieces, approximately 6 × 6 × 6 mm, and were uniformly distributed in several trays and dehydrated at 60 °C using an experimental air dryer (BPG-9140A, Bluepard, China). Acetophenone-D5 (99 atom% D) was purchased from Aladdin (Shanghai, China), and n-alkanes (C7-C30 in hexane) standard solution was purchased from Sigma-Aldrich (St. Louis, MO, USA). Moisture content and volatile compounds were analyzed every 30 min under constant drying conditions during 0–4 h of the drying process. Between 4–12 h of the drying process, moisture content and volatile compounds were analyzed every two hours at constant drying conditions. Dried *L. edodes* were sealed and analyzed within 8 h. Moisture content was determined by removing moisture at 105 °C using an air drying oven (DHG-9053A, Bluepard, China), and was calculated by the weight loss as a percentage of the initial weight.

### 2.2. PT-GC-MS Analysis

Volatile compounds were extracted using an Atomx Purge and Trap Autosampler (Teledyne Tekmar, OH, USA). Samples (dry weight 0.5 g) of different drying times were placed into a 50 mL glass vial. Acetophenone-D5 solution (20 μL, 50 μg/mL) was added as an internal standard (IS). Each sample was preheated for 5 min at 40 °C and then purged for 40 min at 20 °C (purge flow 40 mL/min). The trap was then dry-purged for 2 min at 20 °C (dry purge flow 100 mL/min), followed by desorption for 2 min at 250 °C, and baked for 10 min at 260 °C.

Volatile compounds from PT extract samples were analyzed in triplicate using an Agilent 7890B gas chromatograph equipped with an MSD 5977A mass spectrometer (Agilent technologies co., Ltd., Santa Clara, CA, USA). Volatiles were separated using a 5% phenylmethyl silicone-bounded phase-fused silica capillary column (HP-5ms Ultra Inert 30 × 0.25 × 0.25 μm). The oven temperature program was set for 3 min at 35 °C, raised to 280 °C at 5 °C/min, and held for 10 min. Helium was used as the carrier gas at a flow rate of 1 mL/min. MS determinations were performed in full scan mode (m/z scan range of 35–400 Da). The electron ionization source temperature was maintained at 230 °C, and mass spectra were obtained by electronic impact at 70 eV. Quadrupole temperature was 150 °C.

Volatile compounds were identified based on the comparison of Kovats retention index (RI) and the MS fragmentation patterns with mass spectra in the NIST14 and Wiley11 library. RI values for all studied compounds were calculated using a series of n-alkanes (C7–C30) injected under the same chromatographic conditions [15]. Only compounds whose RI value and MS spectrum were both matched were reported here. Semi-quantitative analysis was based on the internal standard (acetophenone-D5), and the calculation concentration = (At/Ad) × (Md/Mt), where At is the peak area of a particular component in the sample, Ad is the peak area of the internal standard, Md is the weight of internal standard, and Mt is weight of *L. edodes* after t hours of drying.

### 2.3. Sensory Eevaluation

Quantitative Descriptive Analysis (QDA) was employed for the sensory evaluation. The sensory analysis was performed by ten panelists (five males and five females, aged 22–26), trained as described previously [16]. First, the establishment of the vocabulary was completed. All sensory panelists were required to carry out a preliminary sensory evaluation of the sample with their familiar vocabulary. Secondly, the group leader wrote the words on the bulletin board for discussion, revisions, deletions, and additions, as well as the definition of each descriptive word selected. This process was repeated to establish the sensory descriptive words, knowing that all sensory panelists recognized the selected vocabulary. Lastly, each sample was stored at room temperature in a 50 mL glass bottle with a Teflon cover and was provided to a panelist in a sensory room (at 25 °C). Each sample was analyzed in triplicate and opened immediately before the sensory descriptive analysis. Samples were evaluated at different timepoints of the drying process, using six sensory attributes (mushroom flavor, earthy smell, garlic smell, rotten egg flavor, sauté smell and burnt smell) and intensity ratings. Intensity ratings were measured from 0 to 5 (0 = ‘undetected’, 1 = ‘weak’, and 5 = ‘strong’).

### 2.4. Electronic Nose Analysis

A PEN3 electronic nose system (Airsense, Schwerin, Germany) consisting of an array of 10 metal oxide gas sensors (W1C, W5S, W3C, W6S, W5C, W1S, W1W, W2S, W2W, and W3S) was used to estimate the changes in the aroma characteristics of *L. edodes* during hot air drying. Samples with a dry weight of 0.5 g were placed in 40 mL sealed vials and incubated at 50 °C for 10 min before analysis. The headspace gas was pumped into the sensor chamber using a Teflon tubing connected to the needle, with clean air (400 mL/min) as the carrier gas. Response data were acquired at 1 s intervals, over 100 s duration. The maximum resistance changes of each sensor were used for data analysis.

### 2.5. Statistical Analysis

All experiments were conducted in triplicate. The measured data were analyzed using PCA and PLSD-DA by the MetaboAnalyst 3.0 [17]. Significance between related samples was analyzed according to the one-way ANOVA test at the level of 0.05 (*p* < 0.05) using the SPSS 17.0 (Chicago, IL, USA).

## 3. Results

### 3.1. Moisture Content

**Figure 1:**
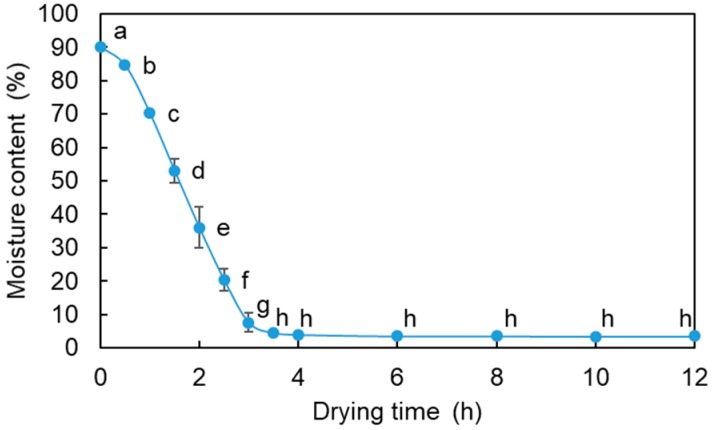
Moisture content of *L. edodes* at different drying times. Means with different letters are significantly different from others (*p* < 0.05).

Fresh *L. edodes* were dried at 60 °C for different times. Moisture changes in *L. edodes* were shown in Figure 1. The moisture content of dried *L. edodes* decreased from 90.19% to 3.50% after drying for 12 h. However, after only three hours of drying, the moisture content was 7.62%. The moisture content of *L. edodes* did not change significantly between 3, 5 and 12 h.

### 3.2. Sensory Evaluation

**Figure 2:**
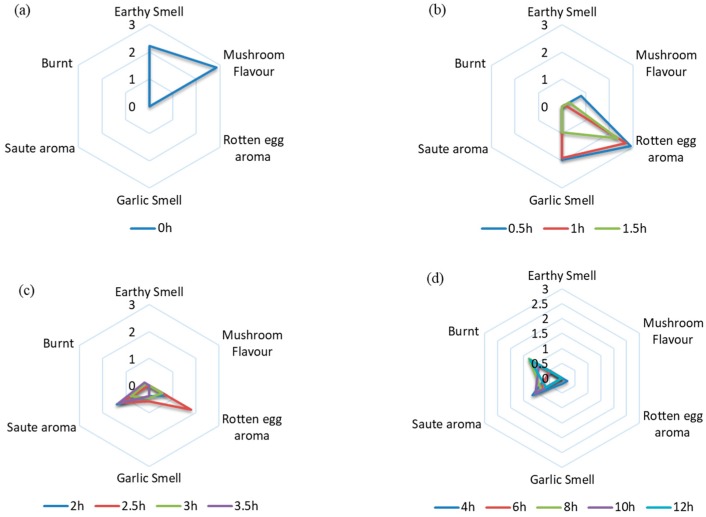

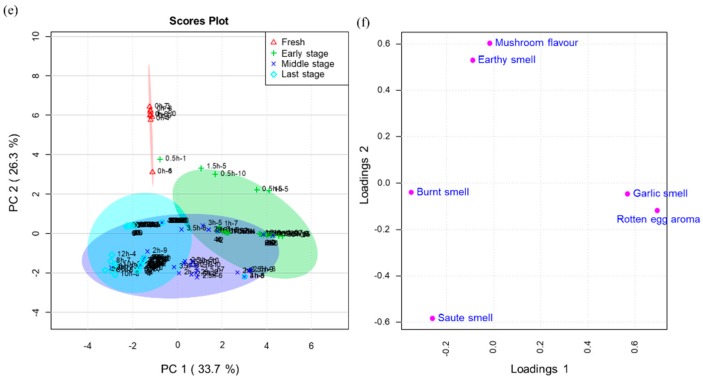
(**a**–**d**) radar fingerprint chart of sensory evaluation for *L. edodes* at different drying times; (**e**) principal component analysis (PCA) scores scatter plot; (**f**) Loading plot of PCA.

According to the different sensory descriptions of *L. edodes* at different drying stage, the aroma of *L. edodes* changed noticeably (Figure 2). It can be seen that the aroma formation of the *L. edodes* during hot air drying can be divided into three stages. Fresh mushrooms have an obvious mushroom aroma and earthy smell. In the early stage (0.5–1.5 h drying time) of the drying process (Figure 2b), the sensory evaluation revealed strong garlic and rotten egg aromas. The next stage (2–3.5 h drying time) was the development of a sauté aroma which moved away from the rotten egg description (Figure 2c). Finally, in samples from 4–12 h drying time, the sauté aroma became more burnt in description (Figure 2d).

Principal component analysis (PCA) is a multivariate technique used to analyze data in which observations are described by several inter-correlated quantitative dependent variables [18]. The sensory evaluation data were analyzed using PCA to underline the differences in the aroma characteristics in *L. edodes* at different drying times. The PCA of sensory evaluation in *L. edodes* at different drying time are shown in Figure 2e,f. PC1 represents 33.7% of the total variance and PC2 represents 26.3% of the total variance. Aromatic profiles of *L. edodes* at different drying times can be divided into four groups according to the location of plotted data. Fresh mushrooms have an obvious mushroom flavor and earthy smell and belong to the first group. The second group contained 0.5–1.5 h dried samples, and was associated with a strong and pungent garlic smell, and a rotten egg flavor. The third group included 2–3.5 h dried samples with sauté smell. The fourth group included 4–12 h dried samples with a burnt smell.

### 3.3. Electronic Nose

**Figure 3:**
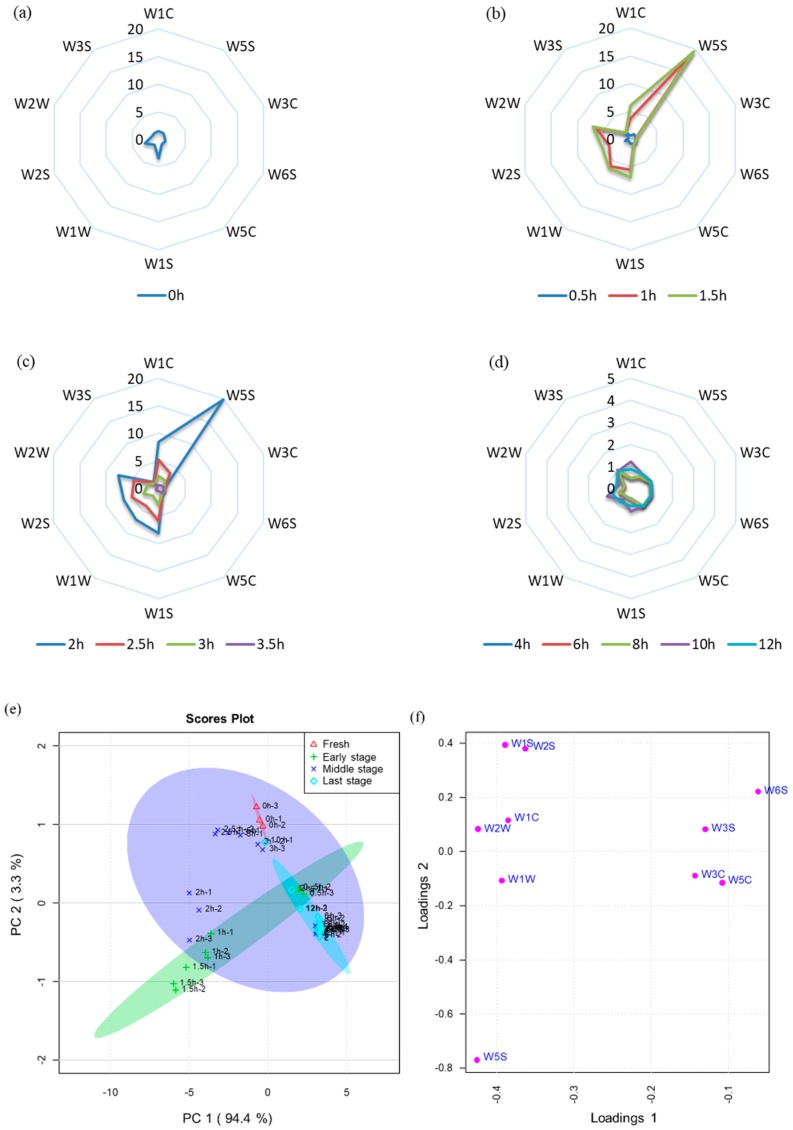
(**a**–**d**) radar fingerprint of electronic nose in *L. edodes* at different drying times; (**e**) PCA scores scatter plot; (**f**) Loading plot of PCA.

The comprehensive flavor characterization of *L. edodes* was detected using an electronic nose equipped with ten types of sensor, which were able to identify and quantify odor compounds. The electronic nose sensor intensity curves of volatile compounds are shown in Figure 3.

The differences observed in electronic nose profiles suggested the noticeable alteration in volatile compounds in *L. edodes* during the drying process (Figure 3a–d). The relative values of W1S and W2S for fresh *L. edodes* were higher than the dried samples and decreased with increasing drying time. The metal oxide gas sensor W1S is most sensitive to short-chain hydrocarbons, while W2S is sensitive to alcohols, aldehydes, and ketones. The relative values of W3S (which is sensitive to alkanes) for 0.5 h-dried *L. edodes* samples were similar to fresh *L. edodes* and other drying times. The radar graph of *L. edodes* aroma at the 1, 1.5, and 2 h drying times almost overlapped, which indicates similarity in volatile compounds, and the relative values of W5S (which is more sensitive to nitroxides) for this drying process were higher than for other drying times.

Sensor W2W was most stimulated at 1–2 h drying time. The radar graph of *L. edodes* aroma at 2.5 and 3 h drying times follows a similar trend, potentially showing that these drying times produce similar volatile compounds. The sensor W3S is sensitive to alkanes, and the *L. edodes* aroma at 3.5, 4, 6, 8, 10, and 12 h drying times also have radar graph profiles higher than other drying times.

The electronic nose data were analyzed using PCA to underline the differences in the volatile compounds in *L. edodes* at different drying times (Figure 3e). The higher the contribution rate, the better the principal components reflect the original multi-index information. The first two principal components (PCs) were extracted by PCA. PC1 represents 94.4% of the total variance, and PC2 represents 3.3% of the total variance (Figure 3e).

### 3.4. PT-GC-MS Analysis of Volatile Compounds

**Figure 4:**
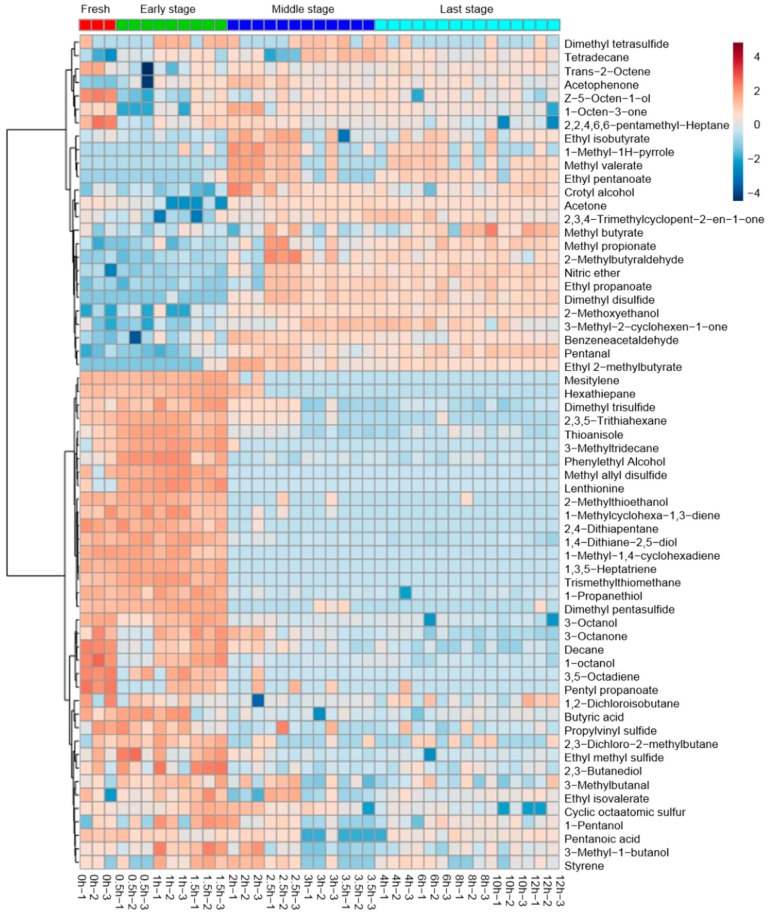
Clustering Heatmap of the concentration of volatile compounds in *L. edodes* at different drying times.

Figure 4 shows the changes in volatile profiles across the fresh and dried *L. edodes*. A total of 62 major volatile compounds were identified.

**Figure 5:**
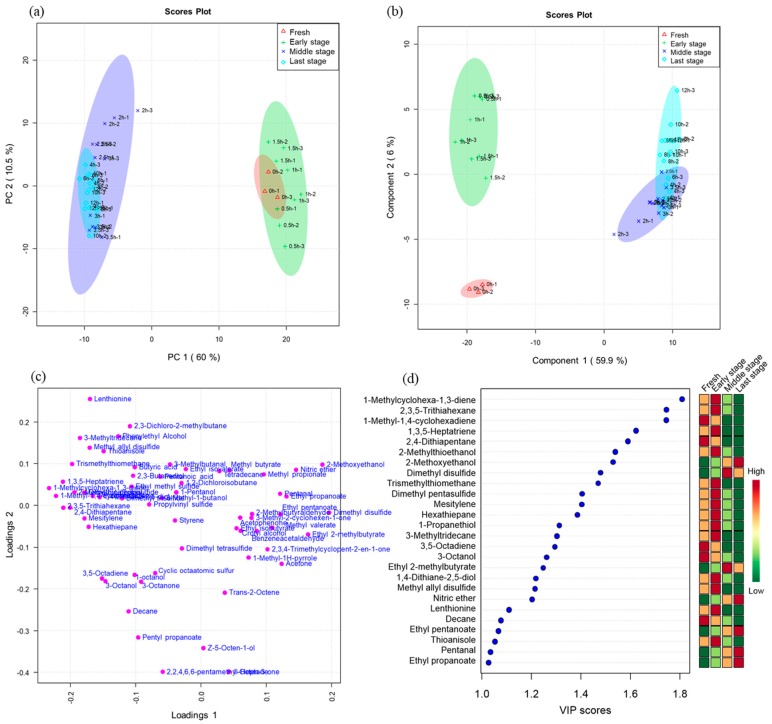
PCA of purge and trap combined with gas chromatography-mass spectrometry (PT-GC-MS) data for *Lentinus edodes* at different drying times. (**a**) PCA scores scatter plot. (**b**) Partial least-squares discriminant analysis (PLS-DA) scores scatter plot. (**c**) Loading plot of PLS-DA. (**d**) Important volatiles (VIP > 1) identified by PLS-DA. The colored boxes on the right indicate the relative concentrations of the corresponding volatiles in different drying stages.

The PCA scores plot is shown in Figure 5a. There was a significant difference between the early stage and the middle stage, but the groups of fresh *L. edodes* samples and the early stage samples were not completely separated. The middle stage samples and the last stage samples were also overlapped. Unlike PCA, PLS-DA is a supervised method that uses multivariate regression techniques. Fresh *L. edodes* samples are clearly separated from other groups by PLS-DA (Figure 5b).

In the fresh group, alcohols were the most important chemical family, followed by ketones and sulfur compounds. The characteristic compounds were 3-octanol and 3-octanone.

The samples from the 0.5 and 1.5 h drying process are mainly characterized by their high sulfur compound content (Figure 5c). Characteristic compounds include dimethyl trisulfide, thioanisole, 2,3,5-trithiahexane, and cyclic sulfur compounds.

The samples from the 2 and 3.5 h drying process generally present a high esters content (Figure 5c). Characteristic compounds include methyl butyrate, ethyl propanoate, ethyl isobutyrate, ethyl 2-methylbutyrate, and ethyl isovalerate.

The samples from 4 and 3.5 h contained mainly aromatic compounds, aldehydes, esters, acids and hydrocarbons. Characteristic compounds included benzaldehyde, nonanal, propanoic acid, 2-methyl-ethyl ester, butanoic acid 2-methyl-ethyl ester, butanoic acid 3-methyl-ethyl ester, p-xylene, 1,3-dimethylbenzene, o-xylene, acetic acid, 3-methyl-undecane, dodecane, tridecane, 3-methyl-tridecane, and tetradecane.

## 4. Discussion

### 4.1. Changes of the Aroma Profiles

Electronic nose systems have been successfully used to detect the characteristic changes in the flavor profiles in food by imitating the human senses through the use of a sensor array and a pattern-recognition system [19]. It is an important method in the rapid detection of chemical components and sensory attributes [9]. According to the sensory and electronic nose analysis, the drying processes of *L. edodes* led to differences in aroma profiles related to the duration of drying. In particular, early (0.5–1.5 h) and late (4–12 h) stages of the process were distinguishable both by sensory and electronic nose analysis, and intermediate drying times resulted in intermediate aromatic profiles. GC-MS results corresponded well to the results of the electronic nose, showing that the outputs of W1S (sensitive to short-chain alkanes) and W2S (sensitive to alcohols, aldehydes and ketones) for fresh *L. edodes* were higher than for the dried samples and decreased with increasing drying time. The PAC showed that volatile compounds of *L. edodes* at fresh and different drying stages were largely overlapping. However, the coherent of clusters corresponding to different drying times indicated that: (1) volatile emissions start changing soon after the drying process; (2) short time drying (1–2 h) is different from long drying; (3) no obvious changes occurred from 4 to 12 h drying. These results indicated that the fingerprint of the Electronic nose data was available to distinguish *L. edodes* samples from different drying stages.

### 4.2. Changes of the Volatile Compositions

In fresh samples, alcohols were the highest peak intensity chemicals, followed by ketones and sulfur compounds. 3-octanone was found in the highest concentration (sweet, fruity, earthy, cheesy and mushroom odor), followed by 3-octanol (sweet, oily and nutty odor). 1-Propanethiol has the highest concentration in the fresh sample compared to other drying stages. However, the drying process resulted in the severe loss of these alcohols and ketones, possibly due to evaporative losses during the drying process.

In the early stages of drying (0.5–1.5 h), the concentration of sulfur compounds increased, including dimethyl trisulfide, thioanisole, 2,3,5-trithiahexane, lenthionine and cyclic sulfur compounds, specifically lenthionine. It is generally speculated that the presence of hydrogen sulfide and sulfur dioxide, and other odorous sulfur compounds can cause a sulfur perception, and thus a negative impression of food [5]. Linear sulfur compounds give a unique aroma of fresh and dried *L. edodes* [2]. Of those volatile sulfur compounds detected, dimethyl trisulfide (fresh onion odor) was found in the highest concentration, followed by thioanisole and 2,3,5-Trithiahexane, which would explain the “garlic flavor” described in sensory evaluation during the early drying stage samples. 1,2,3,5,6-pentathiepane (commonly known as lenthionine) was only detected in the early stage of drying.

In the middle stages of drying (2–3.5 h), the concentration of esters increased (such as methyl butyrate, ethyl propanoate, ethyl isobutyrate, ethyl 2-methylbutyrate and ethyl isovalerate), and the concentration of sulfur compounds decreased (such as 2-(methylthio)ethanol, 2,4-dithiapentane, methyl allyl disulfide, tris(methylthio)methane, dimethyl pentasulfide and lenthionine). The overlapping of radar fingerprints from the 2.5- and 3-h-dried *L. edodes* samples reflects the similarity among the volatile compound emissions from those samples, which were identified by GC-MS mainly as ketones, alcohols, esters and sulfur compounds. An increase in the concentration of esters, acids, and hydrocarbons was found when the drying time was extended to 2.5 h, which may be due to the ester degradation and cleavage reaction of alkoxy radicals caused by the drying process [20]. The content of straight-chain sulfur compounds decreased rapidly during drying, presumably due to their low boiling points [21], or the conversion/ polymerization of these compounds to produce cyclic sulfur compounds [22].

Within the final drying stage (4–12 h), the concentration of sulfur compounds decreased (e.g., 2-(methylthio)ethanol, 2,4-dithiapentane, methyl allyl disulfide). An increase in dimethyl disulfide was also observed during this drying period. The concentration of aromatic compounds and hydrocarbon increased, the production of heterocyclic and aromatic polymers in *L. edodes* was potentially due to the Maillard reaction and protein degradation. The Maillard reaction has been proven to promote the generation of hydrocarbons, heterocyclic and aromatic compounds such as styrene and mesitylene [23]. The sauté and burnt smell in the final drying stage may be mainly due to the acceleration of caramelization and the Maillard reaction, which have been proven to promote the generation of hydrocarbons, heterocyclic and aromatic compounds [23].

Variable importance in projection (VIP) is a weighted sum of squares of the PLS loadings, taking into account the amount of explained Y-variation in each dimension. The important volatiles (VIP > 1) identified by PLS-DA were listed in Figure 5d. Dried *L. edodes* have been especially prized since ancient times in comparison to fresh samples, mainly because of their characteristic smell due to sulfur-containing heterocyclic compounds [22,24]. The concentration of straight-chain sulfur compounds increased first and then decreased rapidly during drying, including 2,4-dithiapentane and dimethyl trisulfide. The reason for this change is most likely their volatilization. Cyclic sulfur compounds increase in concentration over drying time and include such compounds as lenthionine and cyclic octaatomic sulfur, all of which were not detected in fresh *L. edodes*. 1,2,3,5,6-pentathiepane, known as lanthionine, is produced from lentinic acid. The reaction is catalyzed by γ-glutamyl transpeptidase (γ-GTP) and cysteine sulfoxide lyase, followed by the nonenzymatic polymerization of the resulting thiosulfinate [25]. Within the sensory and electronic nose data, the appearance of sulfur-containing compounds, such as dimethyl trisulfide and cyclic sulfur compounds, specifically lanthionine, coincide with an “egg” and garlic aroma. This aroma was absent from fresh shiitake mushroom samples. We can conclude that the drying process plays a crucial role in the production of such odorous compounds.

### 4.3. Emission of the Sulfur-Containing Heterocyclic Compounds

**Figure 6:**
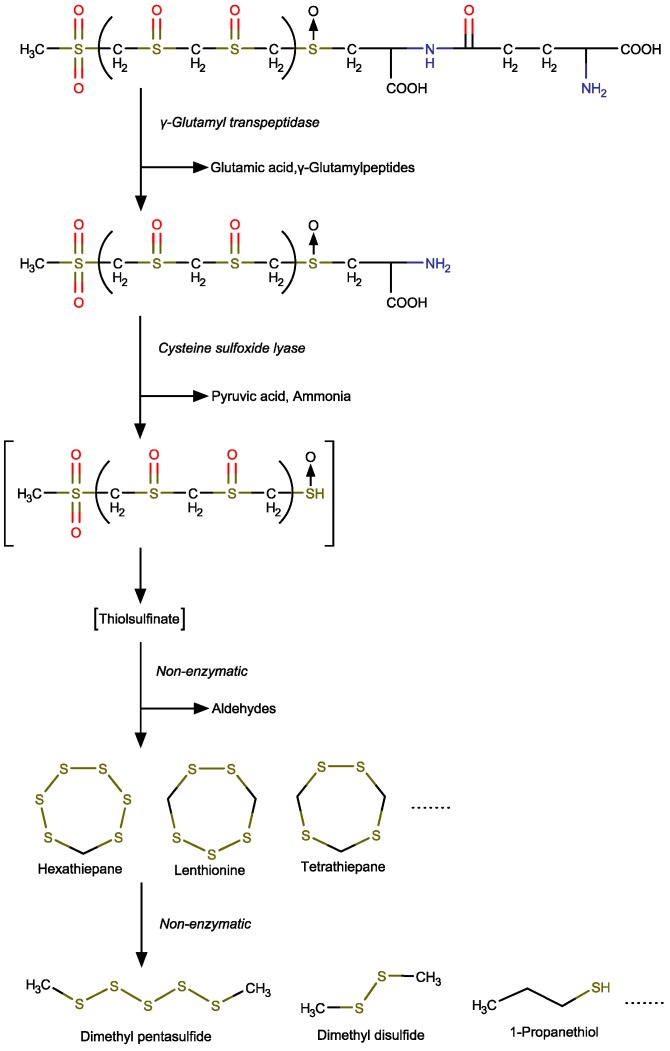
Mechanism of sulfur-containing heterocyclic compound changes through different enzyme-catalyzed and non-enzymatic reactions.

It has been reported that activation of the enzyme γ-GTP under increased temperatures is the reason for the formation of sulfur compounds associated with garlic odor in the early stages of the drying process [26]. Lenthionine could decompose and convert to hydrogen sulfide and thiocarbonyl compounds [22]. As shown in Figure 6, the proposed mechanism of sulfur-containing heterocyclic compound changes includes different enzyme-catalyzed and non-enzymatic reactions: (1) conversion of the lentinic acid to desglutamyl lentinic acid, (2) enzymatic hydrolysis of the resultant desglutamyl compound to yield pyruvic acid, ammonia, and thiolsulfinate, (3) non-enzymatic reaction to form lenthionine and other sulfur-containing heterocyclic compound, and (4) degradation of sulfur-containing heterocyclic compounds to produce the thioether compound.

## 5. Conclusions

Volatile compounds in *L. edodes* at different drying times could be discriminated according to three stages: early stage (<2 h), middle stage (2–3.5 h) and late stage (>3.5 h). Fresh mushrooms have an apparent mushroom flavor and earthy smell which is characterized by 3-octanol and 3-octanone. The early stage revealed a strong garlic and rotten egg odor, which is mainly due to the emission of dimethyl trisulfide, thioanisole, 2,3,5-trithiahexane, and cyclic sulfur compounds. The middle stage developed a sauté aroma, while the rotten egg odor disappeared. In the last stage, the aroma changed from sauté to burnt, which was mainly due to esters and aromatic compounds from caramelization and the Maillard reaction. Electronic nose data showed a similar pattern with the sensory analysis and GC-MS analysis, which was able to distinguish *L. edodes* samples in different drying stage and could be used to assess product quality.
